# Supplementary material for: Dickkopf-Related Protein 1 as Response Marker for Transarterial Chemoembolization of Hepatocellular Carcinomas
Source: Cancers (Basel). 2022 Sep 30;14(19):4807. doi: 10.3390/cancers14194807 (PMC9563450; doi:10.3390/cancers14194807)
Supplement: Supplementary file 1 [file cancers-14-04807-s001.zip › cancers-1899382-supplementary.pdf]

Supplemental Tables

Table S1. Bivariate correlations of DKK-1 as well as AFP and different serum markers at baseline.

**Serum marker**	**correlation with DKK-1**	**p-value**	**correlation with AFP**	**p-value**
AFP	r = 0.022	0.832	n.a.	n.a.
Albumin	r = 0.294	0.003	r = -0.264	0.009
ALT	r = 0.045	0.658	r = -0.096	0.349
Bilirubin	r = -0.329	<0.001	r = 0.0085	0.414
CRP	r = 0.125	0.238	r = 0.198	0.059
Leukocytes	r = 0.581	<0.001	r = 0.228	0.025
Neutrophils	r = 0.598	<0.001	r = 0.231	0.060
Platelets	r = 0.802	<0.001	r = 0.053	0.605
r = Pearson correlation coefficient of r; n.a.=not applicable				

Table S2. Univariate and multivariate logistic regression analyses for response after TACE. We used the forward stepwise logistic regression (likelihood ratio).

**Parameter at baseline**	**Univariate analysis**				**Multivariate analysis**			
	**RC**	**SE**	**OR (95% CI)**	**p value**	**RC**	**SE**	**OR (95% CI)**	**p value**
DKK-1 (pg/mL)	-0.001	<0.001	0.999 (0.998-1)	0.006	-0.001	<0.001	0.999 (0.999-1)	0.010
Male sex	0.624			0.430	-			
Age (years)	0.549			0.459	-			
Liver cirrhosis	1.433			0.231	-			
AFP (ng/mL)	0.136			0.712	-			
AFP-L3 (%)	-0.023	0.01	0.997 (0.958-0.997)	0.027	-0.027	0.012	0.973 (0.951-0.997)	0.025
DCP (ng/mL)	3,84			0.05	-			
Albumin (g/L)	2.374			0.123	-			
ALT (µkat/L)	-1,268	0.472	0.281 (0.112-0.71)	0.007	-1.717	0.612	0.18 (0.054-0.596)	0.005
Bilirubin (µmol/L)	1.456			0.228	-			
CRP (mg/L)	0.097			0.756	-			
Leukocytes (exp^9/L)	2.508			0.113	-			
Neutrophils (exp^9/L)	0.689			0.407	-			
Platelets (exp^9/L)	-0.006	0.002	0.994 (0.989-0.999)	0.013	0.014			0.906
GGT (µkat/L)	0.7165			0.398	-			

CTP								
A	0.077			0.782	-			
B	0.005			0.941	-			
C			REF		-			
BCLC stages								
A	4.468			0.035	-			
B	2.514			0.113	-			
C/D			REF		-			
ECOG performance status								
0	0.001			0.975	-			
1	0.017			0.897	-			
2			REF		-			
Primary tumor site								
Single lobe	0.075			0.784	-			
Bilobar			REF		-			
Number of tumor lesions	1.805			0.179	-			
Total tumor diameter	-0.158	0.061	**0.854 (0.758-0.961)**	**0.009**	0.024			0.878
Extrahepatic HCC at baseline	1.584			0.208	-			

AFP: alpha-fetoprotein, ALT: alanine aminotransferase, BCLC: Barcelona Clinic Liver Cancer score, CI: confidence interval, CRP: C-reactive protein, CTP: Child–Turcotte–Pugh, DCP: des-gamma-carboxy prothrombin, ECOG: Eastern Cooperative Oncology Group, GGT: gamma-glutamyl transpeptitase, HCC: hepatocellular carcinoma, HR: hazard ratio, RC: regression coefficient, REF: reference, SE: standard error, TACE: transarterial chemoembolization.

Supplemental Figures

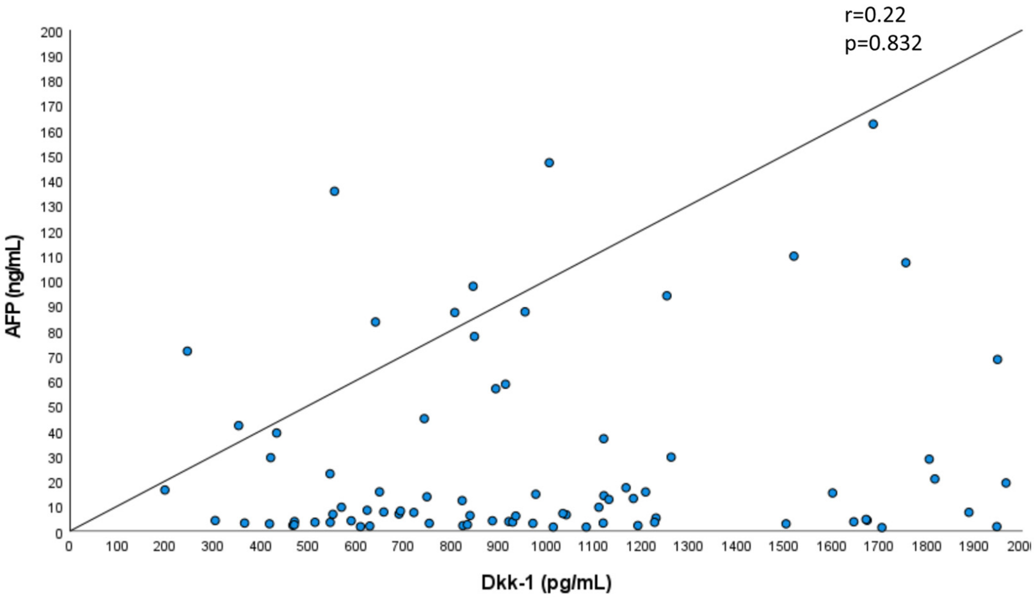

Figure S1. Correlation of DKK-1 and AFP serum levels before TACE in the overall study population (n=97).

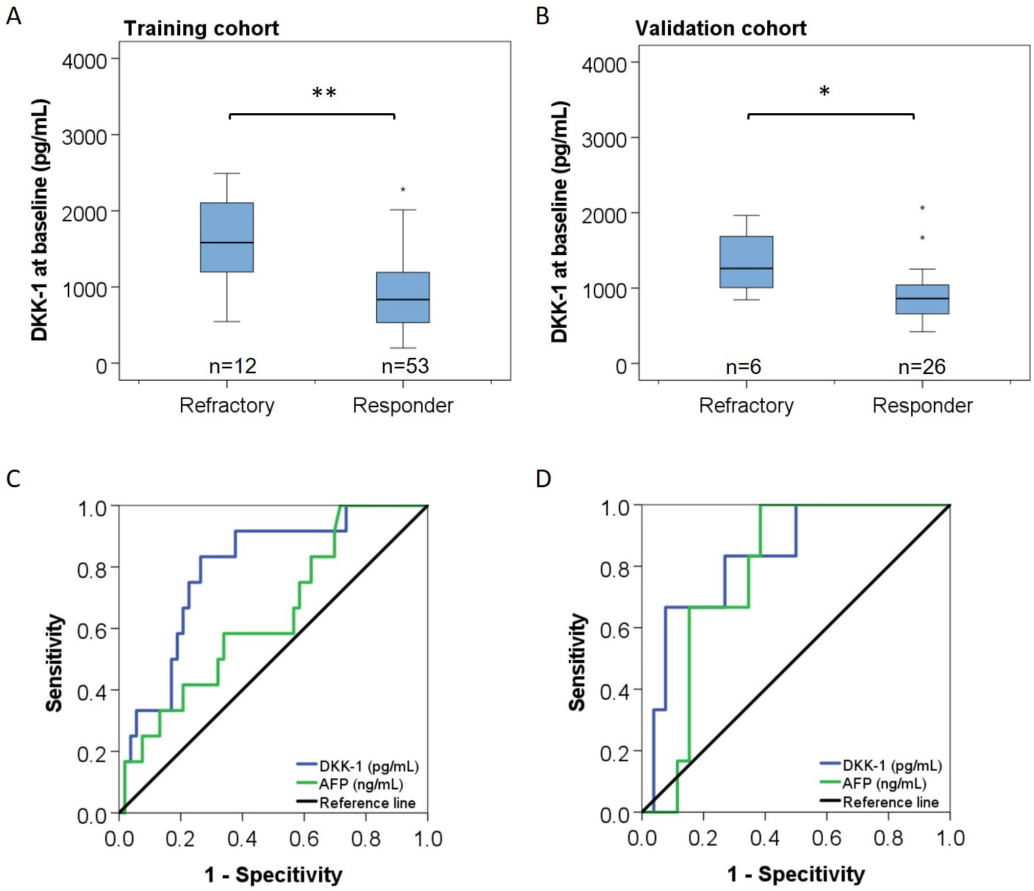

Figure S2: For validation of the predictive response value of DKK-1, our cohort was randomly divided into a training cohort ((A,C) n = 65) and an internal validation cohort ((B,D) n = 32) by random sampling at a ratio of 2:1. *= $p < 0.05$; ** = $p < 0.01$.
